# Does current ovarian endometrioma increase the time for DOR patients to reach live birth in IVF?

**DOI:** 10.1186/s12884-022-04670-7

**Published:** 2022-04-15

**Authors:** Yu Deng, Zhanhui Ou, Minna Yin, Zhiheng Chen, Shiling Chen, Ling Sun

**Affiliations:** 1 Center of Reproductive Medicine, Department of Obstetrics and Gynecology, Nanfang Hospital, Southern Medical University, 510515 Guangzhou, Guangdong PR China; 2 Center of Reproductive Medicine, Guangzhou Women and Children’s Medical Center, Guangzhou Medical University, 510623 Guangzhou, Guangdong PR China

**Keywords:** Ovarian endometriomas, Diminished ovarian reserve, In vitro fertilization, Cumulative live birth rate, Time to achieve live birth

## Abstract

**Background:**

The contents of ovarian endometrioma (OMA) such as inflammatory mediators, reactive oxygen species, and iron may disrupt normal folliculogenesis and result in subsequent oocyte apoptosis. Therefore, women with OMA have a potential risk of diminished ovarian reserve (DOR). The purpose of this study is to compare the in vitro fertilization (IVF) outcomes and efficiency between DOR patients with and without current OMA.

**Methods:**

This retrospective case-control study included a total of 493 women with DOR (serum anti-Müllerian hormone level < 1.1 ng/mL). Ninety patients with OMA (Group A) underwent 191 IVF cycles and 403 patients without ovarian OMA (Group B) underwent 888 IVF cycles in our center between January 2014 and December 2018. Basal characteristics and IVF outcomes were compared between Group A and Group B. Time to achieve live birth were compared between patients with live birth in two groups (Group A1, 31 patients; Group B1, 132 patients).

**Results:**

Clinical and demographic characteristics of patients were similar respectively between groups (A vs. B, A1 vs. B1). There were no statistically significant differences in implantation rate, live birth rate per OPU and per ET cycle and the cumulative live birth rate per patient and per patient with good-quality embryos between Group A and Group B (*P* > 0.05). Total time to achieve live birth has no statistically significant difference between Group A1 and Group B1 (*P* > 0.05).

**Conclusion:**

For DOR women, presence of endometrioma did not affect the IVF outcomes. Even the time to get live birth was not prolonged by current OMA.

## Background

Ovarian endometrioma (OMA) preoperatively diagnosed with transvaginal ultrasound are identified by the presence of a persistent ovarian cyst filled with ground glass echogenic fluid [1, 2]. The contents of OMA such as inflammatory mediators, reactive oxygen species, and iron [3–5] may disrupt normal folliculogenesis and result in subsequent oocyte apoptosis. Therefore, women with OMA have a potential risk of diminished ovarian reserve (DOR). Study on serum anti-mullerian hormone (AMH) level in patients with OMA indicated that endometrioma per se reduced ovarian reserve even before cystectomy [6]. Through damage to pelvic environment and decrease of ovarian reserve, OMA adversely impact women fertility and pregnancy outcomes [7–9]. However, the influence of OMA on assisted reproductive technology outcomes is controversial. Roustan et al. [10] found that both the clinical pregnancy rates and live birth rates per IVF cycle were significantly lower in women with DOR after endometrioma surgery than in idiopathic DOR patients, while a meta-analysis focused on cases with OMA showed inconsistent results [11]. Even with fewer oocytes and MII oocytes retrieved and fewer embryos formed, patients with OMA had similar clinical pregnancy and live birth rates when compared to the control groups.

Most previous studies just focused on the clinical pregnancy rate following a single IVF treatment cycle in DOR patients after ovarian cystectomy, regardless of current presence of OMA at the time of IVF. Since the number of oocytes obtained in a single cycle by DOR patients is limited, multiple cycles are often required to accumulate good quality embryos in clinic. However, there is a lack of research on the cumulative live birth rate and efficiency of IVF cycles for DOR women with presence of OMA.

The purpose of this study was to investigate whether the presence of OMA affected the pregnancy outcomes of multiple IVF cycles and the time to live birth in DOR patients.

## Methods

### Subject recruitment

The retrospective case-control study was conducted in DOR patients aged 20–45 who underwent IVF cycles between January 2014 and December 2018. Because the lack of consensus regarding the diagnostic criteria of DOR, the inclusion criteria in this study were women with an abnormal ovarian reserve test (serum AMH level ≤ 1.1 ng/mL) referring to the Bologna Criteria of poor ovarian response (POR) [12]. All of the IVF and FET cycles were included.

The group of DOR patients with presence of OMA (Group A) included women with current unilateral or bilateral OMA(s) diagnosed by the presence of a persistent round shape, thick-wall cyst > 3 cm in diameter with a low amount of echogenic fluid using transvaginal ultrasound [13], who had not been previously operated. While the group of DOR patients without OMA (Group B) included women with neither current OMA nor a history of ovarian surgery for OMA.

In the two groups, patients were excluded if they had polycystic ovary syndrome (PCOS), untreated hydrosalpinx, genital organ deformity, intrauterine adhesions, recurrent miscarriage, recurrent implantation failure, chromosomal abnormality and autoimmune diseases.

There were 90 patients in Group A undergoing 191 IVF cycles and 403 patients in Group B undergoing 888 IVF cycles.163 patients with live birth were also divided into two groups. Among them, there are 31 patients with OMA (Group A1) and 132 patients without OMA (Group B1).

### Stimulated cycles and frozen embryo transfer

Two types of stimulation protocol were mainly used: luteal long protocol (gonadotropin releasing hormone agonist administration in the luteal phase of the previous cycle) and GnRH-antagonist protocol. The starting gonadotropin dosage was chosen depending on the patients’ baseline characteristics. After 4–5 days of stimulation, the daily dosage of gonadotropin was then adjusted according to ovarian response assessed by the ultrasound follicular measurements and oestradiol levels. Final oocyte maturation was triggered by single injection of 250 mg of human chorionic gonadotropin (hCG, human chorionic gonadotropin, Merck Serono) when the leading follicle reached 18 mm in diameter. Transvaginal ultrasound-guided oocyte retrievals were performed 36 h post hCG administration. Standard fertilization procedures were conducted depended on semen analysis results or prior fertilization condition. Day 3 embryos were evaluated and graded according to the Istanbul consensus [14]. One or two cleavage embryos of the best quality were transferred and remaining embryos were cryopreserved by vitrification for subsequent frozen embryo transfer (FET). Luteal phase was supported by vaginal progesterone gel (Crinone 8%, Merck Serono) 90 mg daily from the day of oocyte retrieval until 10 weekes after conception.

The endometrium was prepared for FET with hormone replacement therapy (HRT). Progesterone supplementation with daily 90 mg vaginal progesterone gel (Crinone 8%, Merck Serono) was administered once the endometrial thickness were more than 8 mm. Luteal phase support continued with vaginal progesterone gel (Crinone 8%, Merck Serono) 90 mg daily and oral dydrogesterone tablets (Duphaston, Abbot) 20 mg per day from the day of embryo transfer to the 10th gestational week.

### Outcome measure

The main outcome was cumulative live birth rate per patient defined as the occurrence of live birth per patient following all ART treatments and time to achieve live birth defined as the duration days from the first day of controlled ovarian stimulation (COH) to the date of live birth.

### Data analysis

All statistical analysis were performed with the assistance of SPSS software (IBM Corp., USA) version 21.0 for Windows. Continuous variables were presented as absolute numbers, mean ± standard deviation (SD), and analyzed by Student’s t test. Categorical variables were presented as percentages and analyzed using the chi-square test or Fisher exact test depending on the sample size. Statistical significance was defined as a two-sided *p* < 0.05.

## Results

### Patients

Demographic and clinical characteristics of patients in two groups are summarized in Table 1. No significant differences were demonstrated between the Group A and Group B in terms of age, antral follicle count (AFC), AMH level, basal serum levels of follicle stimulating hormone (FSH), luteinizing hormone (LH), estradiol (E2), BMI or infertility duration (*P* > 0.05, respectively). However, there are more patients with primary fertility in Group A than that in Group B (38.9% vs 27.5%, *p* = 0.03). Patients in Group A1 had relatively lower AMH level than that in Group B1 (0.6 ± 0.3 vs 0.7 ± 0.3, *p* = 0.02). Other baseline and controlled ovarian hyperstimulation response characteristics of Group A1 and Group B1 were similar.

**Table 1:** Baseline data and controlled ovarian hyperstimulation response characteristics in 493 DOR women with and without endometrioma

	All (*n* = 493)			LB (*n* = 163)		
	Group A Endometrioma	Group B Non- Endometrioma	*P*	Group A1 Endometrioma	Group B1 Non- Endometrioma	*P*
Number of patients	90	403		31	132	
OPU cycles	191	888		55	238	
ET cycles	114	503		45	178	
Age (years)	36.1 ± 5.1	37.7 ± 5.3	0.72	32.6 ± 4.2	34.7 ± 4.3	0.40
AFC	4.3 ± 2.3	4.5 ± 2.1	0.18	5.3 ± 2.2	5.0 ± 2.0	0.49
AMH (ng/ml)	0.6 ± 0.3	0.6 ± 0.3	0.13	0.6 ± 0.3	0.7 ± 0.3	0.02
bFSH (mIU/mL)	9.4 ± 5.9	8.8 ± 5.4	0.47	10.0 ± 6.2	8.2 ± 5.5	0.24
bLH (mIU/mL)	3.4 ± 1.9	3.5 ± 2.2	0.95	3.5 ± 1.6	3.1 ± 1.8	0.88
bE2 (pmol/L)	155.0 ± 87.1	164.9 ± 151.6	0.22	147.8 ± 88.7	161.1 ± 158.2	0.72
BMI (km/m^2^)	21.2 ± 2.8	22.3 ± 2.8	0.86	20.9 ± 3.1	21.5 ± 2.8	0.60
Duration of infertility (years)	3.9 ± 3.0	3.8 ± 3.7	0.21	3.3 ± 2.6	3.2 ± 3.0	0.51
Primary fertility	35/90 (38.9%)	111/403 (27.5%)	0.03	17/31 (54.8%)	54/131 (41.2%)	0.17
Secondary fertility	55/90 (61.1%)	292/403 (72.5%)	0.03	14/31 (45.2%)	77/131 (58.8%)	0.17
range of diameters of endometriomas (cm)	3.0–5.5	–		3.0–4.8	–	
average size of endometriomas (cm)	4.0 ± 0.6	–		3.6 ± 0.4	–	
GnRH-agonist protocol n (%)	26 (13.6)	103 (11.6)	0.44	13 (23.6)	49 (20.6)	0.62
GnRH-antagonist protocol n (%)	162 (84.8)	777 (87.5)	0.32	42 (76.4)	189 (79.4)	0.62
Natural/modified natural n (%)	3 (1.6)	8 (0.9)	0.66	0	0	
Starting dose of gonadotropins (IU)	195.6 ± 70.3	206.8 ± 66.2	0.15	208.4 ± 71.0	223.7 ± 61.6	0.11
Duration of stimulation (days)	8.1 ± 3.4	8.4 ± 3.5	0.89	9.4 ± 3.2	9.0 ± 3.2	0.78
Total doses of gonadotropins (IU)	1730.4 ± 1064.4	1835.7 ± 1040.2	0.66	2168.6 ± 1145.4	2071.8 ± 1015.4	0.18
Serum LH at the day of HCG (mIU/mL)	5.8 ± 6.2	5.4 ± 6.7	0.93	3.7 ± 4.1	3.5 ± 3.4	0.93
Serum E2 at the day of HCG (pg/mL)	3896.5 ± 2631.6	3792.9 ± 2859.0	0.70	4601.2 ± 2671.8	4561.3 ± 3032.7	0.67
Serum P at the day of HCG (ng/mL)	1.7 ± 1.1	1.6 ± 1.1	0.5	1.7 ± 0.9	1.7 ± 1.1	0.07
Endometrial thickness at the day of HCG (mm)	9.3 ± 2.2	8.9 ± 3.0	0.13	10.1 ± 2.7	9.6 ± 2.5	0.65

*Abbreviations*: *LB* Live birth, *OPU* Ovum pick up, *ET* Embryo transfer, *AFC* Antral follicle count, AMH Anti-Műllerian hormone, *FSH* Follicle stimulating hormone, *LH* Luteinizing hormone, *E2* estradiol, *BMI* Body mass index, *GnRH* Gonadotrophin-releasing hormone, *HCG* Human chorionic gonadotropin, *P* Progesterone

### Comparisons of laboratory parameters

Comparisons of laboratory parameters respectively between Group A and Group B, Group A1 and Group B1 are shown in Table 2. No significant differences were present in the mean number of OPU cycles, ET cycles, oocytes retrieved, 2 pronuclear zygotes (2PN), embryos and good-quality embryos per patient and per cycle. In addition, 2PN rate, embryos and good-quality embryo rate (per oocyte) were similar in both groups.

**Table 2:** Comparisons of cycle parameters

	All (*n* = 493)			LB (*n* = 163)		
	Group A Endometrioma	Group B Non- Endometrioma	*P*	Group A1 Endometrioma	Group B1 Non- Endometrioma	*P*
OPU cycle number n (%)						
1	41 (45.56)	172 (42.68)	0.70	19 (61.29)	74 (56.06)	0.74
2	22 (24.44)	107 (26.55)	0.78	5 (16.13)	29 (21.97)	0.64
≥ 3	27 (30)	124 (30.77)	0.99	7 (22.58)	29 (21.97)	0.87
ET cycle number n (%)						
1	51 (68)	225 (65.41)	0.77	21 (67.74)	95 (71.97)	0.8
2	16 (21.33)	89 (25.87)	0.5	7 (22.58)	29 (21.97)	0.87
≥ 3	8 (10.67)	30 (8.72)	0.76	3 (9.68)	8 (6.06)	0.75
OPU cycles per patient	2.1 ± 1.4	2.2 ± 1.5	0.65	1.8 ± 1.2	1.8 ± 1.4	0.72
ET cycles per patient	1.3 ± 1.1	1.3 ± 0.9	0.50	1.5 ± 0.8	1.4 ± 0.6	0.14
Oocytes retrieved per patient	5.3 ± 4.0	5.7 ± 4.5	0.68	6.3 ± 4.2	6.8 ± 4.6	0.51
2PN per patient	3.4 ± 2.9	3.7 ± 2.9	0.35	4.1 ± 2.8	4.6 ± 2.9	0.10
Embryos per patient	3.0 ± 2.6	3.1 ± 2.5	0.34	3.6 ± 2.1	3.9 ± 2.2	1.00
Good-quality embryos per patient	1.2 ± 1.4	1.2 ± 1.2	0.25	1.6 ± 1.2	1.6 ± 1.1	0.91
Embryos transferred per patient	2.3 ± 2.1	2.3 ± 1.8	0.09	2.8 ± 1.8	2.6 ± 1.4	0.41
Oocytes retrieved per cycle	3.0 ± 2.3	3.2 ± 2.7	0.33	4.1 ± 2.1	4.5 ± 3.0	0.13
2PN per cycle	1.9 ± 1.7	2.1 ± 1.9	0.98	2.76 ± 1.64	3.10 ± 2.07	0.41
Embryos per cycle	1.7 ± 1.6	1.8 ± 1.6	0.66	2.42 ± 1.32	2.68 ± 1.67	0.24
Good-quality embryos per cycle	0.7 ± 0.9	0.7 ± 0.9	0.84	1.19 ± 1.00	1.16 ± 0.98	0.74
Embryos transferred per cycle	1.5 ± 0.8	1.6 ± 0.8	0.54	1.85 ± 0.41	1.91 ± 0.47	0.98
2PN%	306/478 (64.0%)	1500/2308 (65.0%)	0.69	128/195 (65.6%)	602/897 (67.1%)	0.69
Embryos% (per oocyte)	267/478 (55.9%)	1256/2308 (54.4%)	0.57	110/195 (56.4%)	518/897 (57.8%)	0.73
Good-quality embryos% (per oocyte)	107/478 (22.4%)	467/2308 (20.2%)	0.29	49/195 (25.1%)	208/897 (23.2%)	0.56

*Abbreviations*: *LB* Live birth, *OPU* Ovum pick up, *ET* Embryo transfer, *PN* Pronuclear zygotes

### Comparisons of IVF outcomes

Table 3 represented the comparisons of IVF outcomes between Group A and Group B. There were no statistically significant differences in the percentage of patients with no embryos transferred and patients with good-quality embryos, implantation rate, live birth rate per OPU and per ET cycle and cumulative live birth rate per patient and per patient with good-quality embryos between Group A and Group B. (*P* > 0.05).

**Table 3:** Comparisons of IVF outcomes

	All (*n* = 493)		
	Group A Endometrioma	Group B Non- Endometrioma	*P*
Patients with no embryos	15/90 (16.7%)	59/403 (14.6%)	0.63
Patients with Good-quality embryos	56/90 (62.2%)	255/403 (63.3%)	0.85
Implantation rate	48/210 (22.9%)	206/935 (22.0%)	0.80
Live birth rate per OPU cycle	31/191 (16.2%)	132/888 (14.9%)	0.63
Live birth rate per ET cycle	31/114 (27.2%)	132/503 (26.2%)	0.84
Cumulative live birth rate per patient	31/90 (34.4%)	132/403 (32.8%)	0.76
Cumulative live birth rate per patient with Good-quality embryos	27/56 (48.2%)	111/255 (43.5%)	0.52

*Abbreviations*: *OPU* Ovum pick up, *ET* Embryo transfer

No statistically significant differences were found in the percentage of patients respectively with only 1 OPU cycle, only 1 ET cycle and FET cycles between Group A1 and Group B1. Total time to achieve live birth were similar in the two groups (*P* > 0.05) (Table 4).

**Table 4:** Comparisons of time to achieve live birth in patients with live birth

	LB (*n* = 163)		
	Group A1 Endometrioma	Group B1 Non- Endometrioma	*P*
Patients with only 1 OPU cycle (%)	19/32 (59.4%)	74/132 (56.1%)	0.73
Patients with only 1 ET cycle (%)	21/32 (65.6%)	95/132 (72.0%)	0.48
Patients with FET cycles %	23/31 (74.2%)	98/132 (74.2%)	1.00
Time to achieve live birth (day)	431.3 ± 167.5	432.8 ± 198.1	1.00

*Abbreviations*: *LB* Live birth, *OPU* Ovum pick up, *ET* Embryo transfer; *FET* frozen embryo transfer

## Discussion

In this study, we found that pregnancy outcomes of multiple IVF cycles were similar in DOR women with or without current endometrioma. Thus, our study revealed that surgery of endometrioma may not be considered in patients with poor ovarian reserve.

Previous studies indicated that surgical management of endometriomas negatively affected ovarian reserve [8, 15, 16], due to unintentional removal of normal tissue during ovarian cystectomy [17, 18]. Raffi and Alborzi reported a significant decrease in AMH level and an increase in FSH after OMA surgery [19, 20]. Furthermore, current evidence suggested that ovarian cystectomy prior to IVF failed to improve IVF outcomes in infertile women with OMA [9, 21, 22]. A recent study reported that poor response to ovarian stimulation and cycle cancellation were more frequent in patients who had previous endometrioma surgery compared to patients with non-operated endometrioma. This study also reported that the pregnancy rates were similar in patients who had previous endometrioma surgery compared to patients with non-operated endometrioma, which was consistent with our study [23]. However, the majority of published studies suggested that ovarian surgery for endometrioma was associated with impaired IVF outcome in DOR patients. A recent retrospective case–control study demonstrated that DOR women by endometrioma cystectomy had significantly lower clinical pregnancy and live birth rates per IVF cycle than women with idiopathic DOR diagnosed by the Bologna criteria [10]. On the contrary, it has been reported that the IVF outcomes were promising even with the presence of ovarian endometriomas [24]. Therefore, surgical excision of endometriomas prior to IVF was not recommended especially for the patients who already have a decreased ovarian reserve.

As a cause of infertility, OMA per se before surgical treatment may also result in DOR through impairing ovarian reserve and altering ovarian functional anatomy [25]. Previously, the impact of current OMAs on IVF outcomes in women with DOR has not been fully investigated. The majority of studies published to date suggested significantly lower clinical pregnancy rates and live birth rates per IVF cycle in women with DOR caused by endometrioma cystectomy than women suffering from tubal infertility, while women with current endometrioma were often excluded [10]. However, our study focused on DOR patients without operated OMA showed inconsistent results. We observed no significant difference in IVF outcomes in the group of DOR patients with presence of OMA compared with group of DOR absent of OMA.

Firstly, our results showed similar mean number of oocytes and embryos per patient and per IVF cycle in the two groups. On the contrary, previous studies and a recent meta-analysis generally showed that un-operated OMA significantly reduced the number of oocytes retrieved, metaphase II oocytes and embryos formed in IVF treatment [11, 26]. Even when the number of oocytes retrieved were adjusted for age and ovarian reserve markers, a retrospective cross-sectional study demonstrated that ovarian response was significantly lower in women with endometrioma than women with other infertility factors undergoing their first ovarian stimulation for IVF/ICSI [27]. Besides, the presence of OMA reduced follicle accessibility and the number of oocytes by hindering follicles at the time of retrieval [28]. Different from previous reports, our case-control study focused on DOR patients and suggested that OMA did not reduce ovarian response and number of oocytes retrieved in women with poor ovarian deserve. In addition, intra-patient comparisons between ovaries with unilateral endometriomas and healthy ovaries of the same individuals suggested the number of oocytes, MII oocytes and embryos were similar [11]. Therefore, ovarian reserve status appears to be a more important confounding factor for oocyte number than the presence of endometrioma alone.

The impact of current OMA on oocyte quality in ART remains debated. Based on two matched case-control studies, [29, 30] the oocyte quality may be negatively affected by endometriosis. However, the results of our study revealed that current presence of OMA was not detrimental for oocyte quality, fertility rate and embryo quality in same condition of DOR. Similar number of good-quality embryos and comparable good-quality embryo rate per oocyte were shown in both groups. This result is consistent with a recent Meta-analysis, which indicated that there was no significant difference in the number of high-quality embryos among the endometrioma compared to the control groups [24, 31].

Based on our data, there were only 11 cases which had an accidental aspiration of endometrioma fluid during oocyte retrieval and no ovarian abscess complications. It was uncommon in DOR patients because we could easily avoid puncture through endometrial cysts during OPU due to the small number of follicles. However, even with the possible event, previous studies found that exposure of oocytes to endometrioma fluid did not affect early embryo development or IVF outcomes [32–34].

Furthermore, another study demonstrated that patients with OMA had similar aneuploidy rates and fertility outcomes compared to healthy controls, suggesting that the embryo quality and endometrial receptivity remained unaffected despite of poor response to ovarian stimulation and a possible decrease in the number of oocytes retrieved [9]. Even with fewer oocytes and MII oocytes retrieved and fewer embryos formed, patients with endometrioma had similar clinical pregnancy and live birth rates when compared to those in control groups [11]. There was no statistically significant difference in cumulative live birth rate per patient with good-quality embryos between two groups, which indicated that endometrial receptivity and environment were unaffected by current OMA. However, there were still a lack of studies assessing the impact of endometrioma on IVF outcome in DOR patients. Since the number of oocytes obtained in a single cycle in DOR patients is limited, multiple cycles are often required to accumulate good quality embryos. However, most previous studies have focused on the clinical pregnancy outcomes following a single IVF cycle in DOR patients after endometrioma cystectomy, regardless of current presence of endometrioma at the time of IVF [35]. Therefore, in order to analyze efficiency of IVF treatments for DOR patients with OMA, we observed all treatment cycles of the same patient and the cumulative live birth rate per patient in our study. The results of multiple IVF treatment cycles in the present study demonstrated that the cumulative live birth rate at the end of multi-cycle IVF treatment was not impaired by presence of OMA in patients with DOR.

Moreover, our strength compared with the previous studies was that we observed all continuous treatment cycles of each patient and calculate the time to achieve live birth, which made it more convincing and powerful to illustrate the efficiency of overall treatment. Therefore, we concluded that current OMA did not aggravate the difficulty of IVF treatment in DOR patients. We suggest that it was ovarian reserve not endometrioma the most important factor for IVF outcome. Therefore, endometrioma cystectomy prior to IVF was not recommended for DOR patients.

Limitations of this study: The main limitation of this study is the relatively lower sample size which may limit the statistical power; besides, the cause of DOR in patients without OMAs may be different; furthermore, the heterogeneity of OMA should also be taken into account including painful symptomatology, size for endometrioma and involvement of one or both sides of the ovary. Although the difference between the backgrounds of two groups were largely reduced and balanced in this case-control study, further prospective randomized controlled trials and a detailed subgrouping are needed to confirm these findings.

## Conclusions

For DOR women, presence of endometrioma did not affect the IVF outcomes of multiple cycles. Even the time to live birth was not prolonged by current OMA.

## Data Availability

The datasets used and/or analyzed during the current study are available from the corresponding author on reasonable request.

## Abbreviations

OMA: Ovarian endometrioma
DOR: diminished ovarian reserve
IVF: In vitro fertilization
AMH: Anti-mullerian hormone
POR: Poor ovarian response
PCOS: Polycystic ovary syndrome
hCG: Human chorionic gonadotropin
FET: Frozen embryo transfer
HRT: Hormone replacement therapy
COH: Controlled ovarian stimulation
SD: Standard deviation
AFC: Antral follicle count
FSH: Follicle stimulating hormone
LH: Luteinizing hormone
E2: Estradiol
2PN: 2 pronuclear zygotes
